# Iron Oxide Nanoparticles Modified with Galloylated DNA for Magnetically Enhanced DNA‐Directed Assembly

**DOI:** 10.1002/advs.202501491

**Published:** 2025-06-29

**Authors:** Murali Golla, Hyunjin Jeon, Shine K. Albert, Hojun Lee, Seulki Kang, Moon Jeong Park, So‐Jung Park

**Affiliations:** ^1^ Department of Chemistry and Nanoscience Ewha Womans University Seoul 03760 Republic of Korea; ^2^ Department of Chemistry Pohang University of Science and Technology (POSTECH) Pohang 37673 Republic of Korea; ^3^ Graduate Program in Innovative Biomaterials Convergence Ewha Womans University Seoul 03760 Republic of Korea

**Keywords:** DNA Conjugation, Iron Oxide Nanoparticle, magnetic interaction, Metal‐Phenol interaction, surface modification

## Abstract

A straightforward, one‐step method is presented for DNA functionalization of iron oxide nanoparticles (IONP) using galloylated DNA through multidentate metal‐phenol interactions. The DNA‐modified IONPs exhibit excellent stability under diverse buffer conditions and display intriguing DNA binding properties, influenced by the superparamagnetic property of IONPs. The DNA denaturation behavior can be categorized into two regimes: the magnetic‐dominant regime and the DNA‐dominant regime. In the magnetic regime, where IONPs are assembled using relatively short DNA linkers, magnetic and DNA interactions jointly stabilize the assemblies, resulting in an unusually weak length dependence of DNA melting temperature. As the linker length increases, the system transitions into the DNA‐dominant regime, where the magnetic effect diminishes, and the assemblies exhibit conventional DNA length‐dependence. This study offers a simple and robust approach for DNA functionalization of IONPs, which can be extended to other metal compound nanoparticles, and highlights the potential of utilizing magnetic reinforcement to modulate DNA‐based nanoparticle assembly.

## Graphical abstract

A simple one‐step method is presented for DNA functionalization of iron oxide nanoparticles (IONP) using multidentate galloylated DNA. The DNA‐modified IONPs exhibit unique DNA binding properties of magnetically enhanced DNA interconnects. This study underscores the importance of nanoparticle (NP) effects in DNA sequence design and highlights the exciting potential of utilizing magnetic forces to regulate biological interactions.

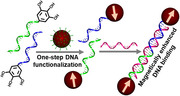

## 1 Introduction

DNA‐modified nanoparticles (NPs) have been widely used in diverse research areas ranging from device fabrications to biomedical applications.^[^ ^1^, ^2^, ^3^, ^4^, ^5^, ^6^ ^]^ The DNA shell surrounding a NP, which is called spherical nucleic acids, exhibits distinct and highly useful molecular recognition properties compared to soluble DNA, such as enhanced DNA binding, sharp melting transitions, high resistance to enzymatic degradation, and efficient cellular uptake.^[^ ^7^, ^8^, ^9^, ^10^, ^11^, ^12^ ^]^ Among various types of NPs, gold nanoparticles (GNP) have been extensively used because they can be easily functionalized with thiolated DNA through the direct thiol‐gold interaction.^[^ ^13^, ^14^, ^15^, ^16^, ^17^ ^]^ Developing similarly simple and efficient surface functionalization methods for other types of NPs could open new opportunities for both materials fabrication and biomedical applications. Superparamagnetic iron oxide nanoparticles (IONP) are particularly intriguing for a number of applications including magnetic separation, medical imaging, and therapeutics.^[^ ^18^, ^19^, ^20^, ^21^, ^22^, ^23^, ^24^ ^]^ Previously reported methods for DNA functionalization of IONPs typically involve multi‐step processes, including the phase transfer of hydrophobic IONPs into water, introduction of functional groups onto the IONP surface, and subsequent DNA coupling. Common approaches adopt NP coating with polymer or silica layers, followed by DNA coupling through various chemical reactions such as alkyne‐azide, acid‐amine, and thiol‐ene reactions.^[^ ^18^, ^21^, ^25^, ^26^, ^27^, ^28^, ^29^ ^]^ While they are based on well‐established chemistry, the multi‐step procedures typically require careful control of reaction conditions. Moreover, thick silica or polymer coatings make the overall size of NPs larger, limiting their use in certain applications. Although single‐step DNA functionalization methods have been reported involving DNA coupled with multiple carboxylates, the complexity of the DNA conjugates limits their universal applicability.^[^ ^22^, ^23^ ^]^

**Figure 1:**
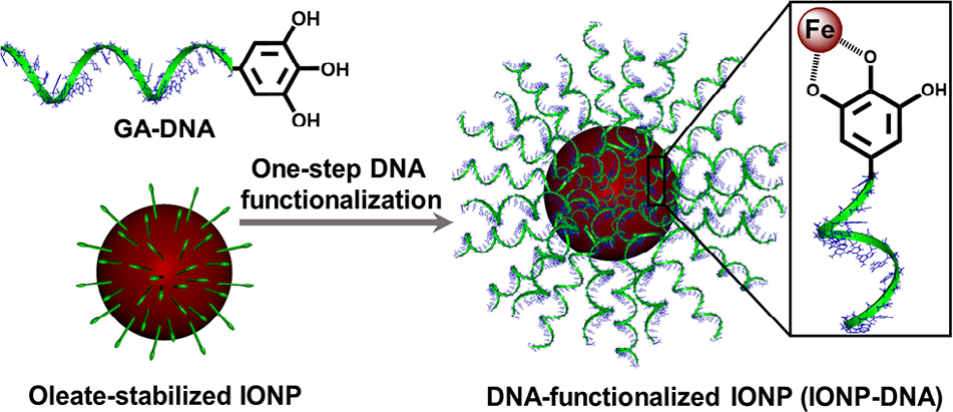
One‐step DNA functionalization of IONP with GA‐DNA.

Here, we present a simple and direct method for DNA functionalization of IONPs, utilizing polyvalent metal‐phenol interactions with galloylated DNA (GA‐DNA) (**Figure** **1**). The strong interaction between gallol and iron, involving chelated Lewis acid‐base coordination and π‐electron/d‐orbital communication, resulted in stable IONP‐DNA conjugates (IONP‐DNA) across various buffer conditions.^[^ ^30^, ^31^, ^32^ ^]^ Furthermore, a systematic investigation into DNA binding properties revealed that the magnetic dipole interaction between IONPs can significantly enhance the stability of DNA‐linked IONPs, suggesting the potential for controlling biomolecular interactions using magnetic forces.

## 2 Results and Discussion

### 2.1 DNA‐Functionalization of IONP

**Figure 2:**
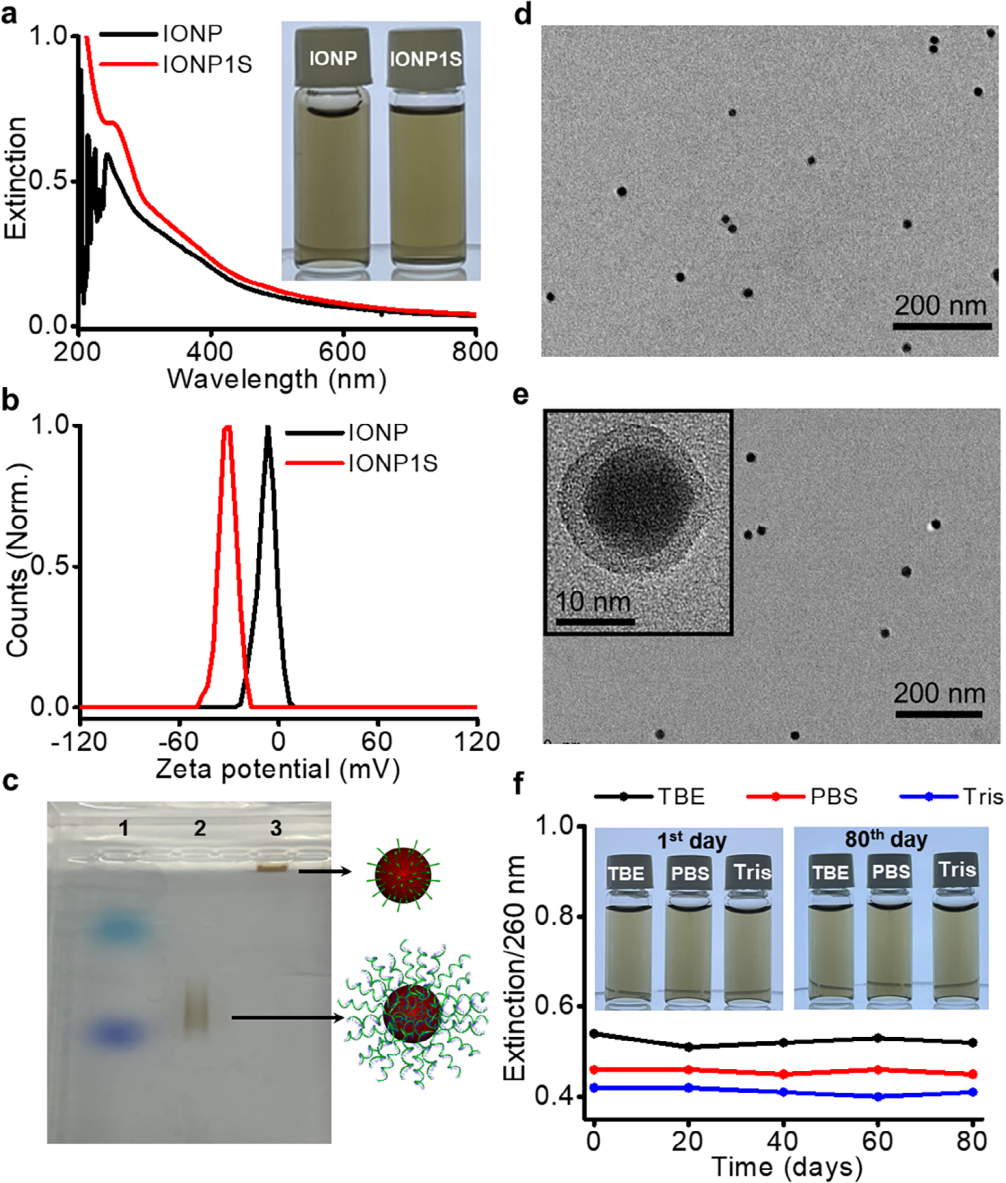
Characterization of IONP‐DNA. a,b) UV–vis extinction spectra (a) and zeta potential analysis (b) of IONP and IONP1S. c) Agarose gel (0.5%) image of tracking dye (lane 1), IONP1S (lane 2), and IONP (lane 3). d,e) TEM images of IONP (d) and IONP1S (e); the inset shows a high‐magnification image of IONP1S stained with UranyLess. f) The extinction at 260 nm of IONP1S dispersed in 0.3 M PBS, TBE, and Tris buffers over 80 days; the inset shows photographs of IONP1S solutions on the 1^st^ (left) and 80^th^ (right) day.

Four GA‐DNA constructs, DNA1, DNA2, DNA1S, and DNA2S were synthesized by reacting amine‐modified oligonucleotides with activated gallic acid. DNA1S and DNA2S include additional polyadenine (A_10_) spacers between the recognition sequence and the gallol anchor. The DNA sequences used in this study are listed in Table S1 (Supporting Information). Detailed synthetic procedures and characterization data are provided in the supporting information (Schemes S1 and S2; Figures S1–S7, Supporting Information). IONPs of two different sizes (13.3 ± 0.9 nm and 17.1 ± 1.3 nm) were synthesized by the thermal decomposition method using oleic acid as the surface ligand.^[^ ^33^ ^]^ In our typical DNA modification procedure, 13 nm IONPs were mixed with DNA1S at a 1:500 molar ratio in 80:20 THF:water mixture. After 5 h, the water content was increased to 50%, and the mixture was left at room temperature for 12 h. Following the evaporation of THF, the salt concentration was adjusted to 0.1 M. After 12 h, the resulting solution was subjected to five freeze/thaw cycles^[^ ^16^ ^]^ to produce stable IONP‐DNA1S conjugates (IONP1S). The number of DNA strands per particle was determined to be 84.1 ± 9 (Figure S8, Supporting Information), which is comparable to that of GNPs modified with thiolated DNA.^[^ ^34^ ^]^ The UV–vis spectra of IONP‐DNA showed the characteristic DNA peak at 260 nm superimposed on the broad absorption spectrum of IONPs (**Figure** **2**a). Zeta potential and dynamic light scattering measurements indicated an increase in negative charge from ‐6.5 to ‐31.5 mV (Figure 2b) with a slight increase in hydrodynamic size (Figure S9e, Supporting Information) upon DNA functionalization. The electrophoretic mobility of IONP1S in agarose gel also supports the successful DNA functionalization (Figure 2c). Additionally, TEM imaging with UranyLess staining confirmed the presence of a DNA shell covering the IONP (Figure 2d,e). Owing to the strong chelation effect of the gallol,^[^ ^35^ ^]^ the conjugates were found to be stable over two months in various conditions, including buffers with metal‐binding molecules such as phosphates (Figure 2f; Figure S10, Supporting Information). The DNA functionalization method was also applied to larger IONPs (17 nm) and various DNA sequences, demonstrating the generality of this approach (Figure S9, Supporting Information).

### 2.2 DNA Binding Properties of IONP‐DNA

**Figure 3:**
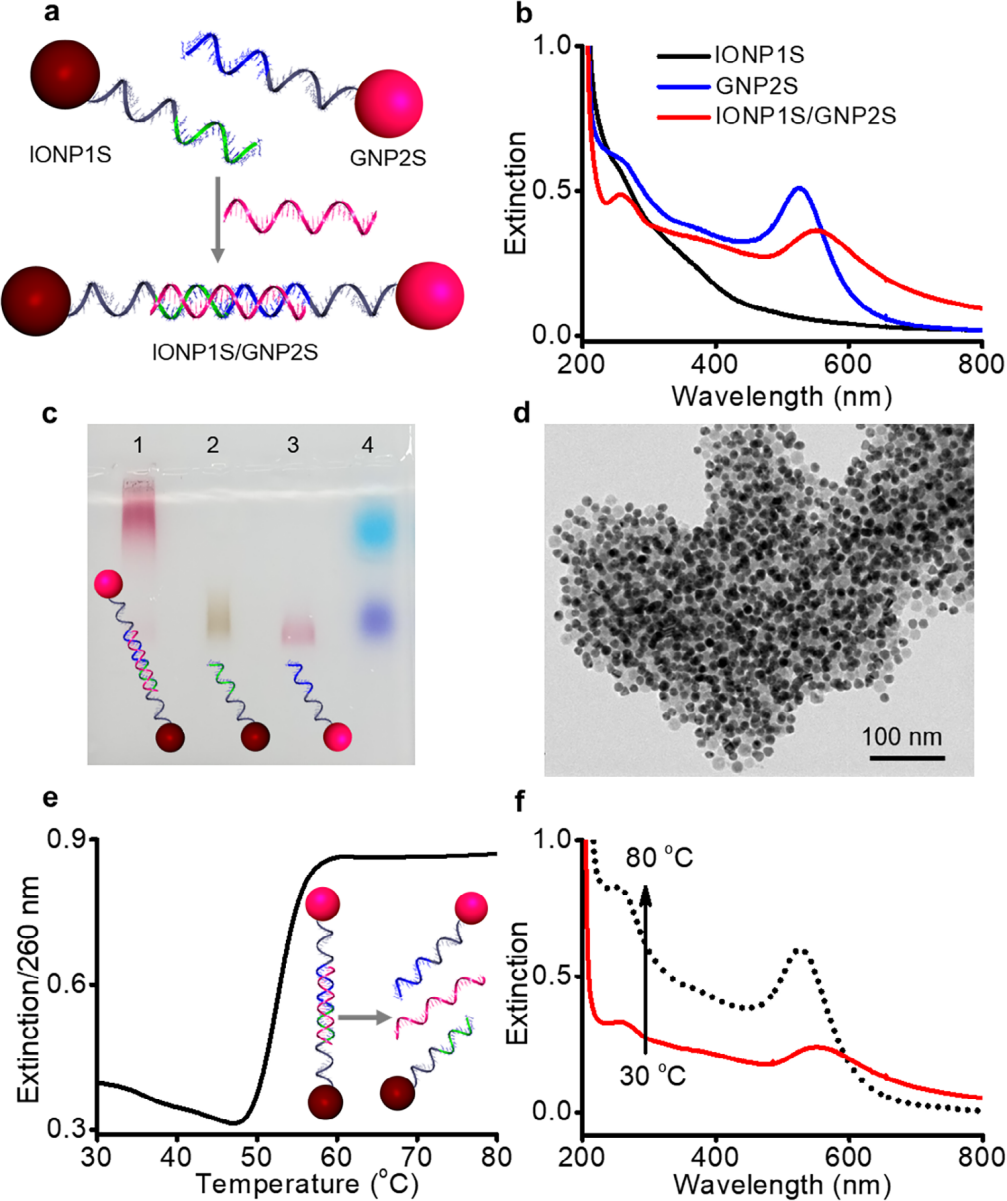
Molecular recognition property of IONP‐DNA. a) Scheme describing L21‐mediated self‐assembly of IONP1S and GNP2S. b) Extinction spectra of IONP1S, GNP2S, and IONP1S/GNP2S. c) Agarose gel (0.5%) image showing IONP1S/GNP2S (lane 1), IONP1S (lane 2), GNP2S (lane 3), and tracking dye (lane 4). d) TEM image of IONP1S/GNP2S. e) Melting profile of IONP1S/GNP2S. f) Extinction spectra of IONP1S/GNP2S at 30 °C and 80 °C.

The molecular recognition properties of IONP‐DNA were examined by hybridizing them with DNA‐grafted GNPs (GNP‐DNA) (**Figure** **3**) prepared using a standard protocol with thiolated DNA (Figure S11, Supporting Information).^[^ ^13^ ^]^ Typically, an equimolar ratio of IONP1S was mixed with GNPs (12.1 ± 0.8 nm) grafted with the thiolated version of DNA2S (GNP2S) in 0.3 M PBS. A linker DNA (L21, 100 equivalents), complementary to the recognition sequences of DNA1S and DNA2S, was added to interconnect the IONPs and GNPs through sequence‐specific DNA interactions (Figure 3a). The DNA hybridization resulted in macroscopic aggregation of IONPs and GNPs. The observed redshift and damping of the surface plasmon resonance (SPR) band of GNPs (Figure 3b) and retarded electrophoretic mobility (Figure 3c) are consistent with TEM data showing extended aggregates of IONPs and GNPs (Figure 3d). Thermal denaturation analysis displayed a sharp dehybridization transition at 53 °C (Figure 3e), characteristic of DNA‐linked NP assemblies. The SPR band returned to that of dispersed GNPs when the temperature exceeded the DNA melting temperature (*T*_m_) (Figure 3f). These results confirm that DNA grafted onto IONPs retains its molecular recognition properties and undergoes efficient DNA hybridization.

### 2.3 NP Effect on DNA Linkages

**Figure 4:**
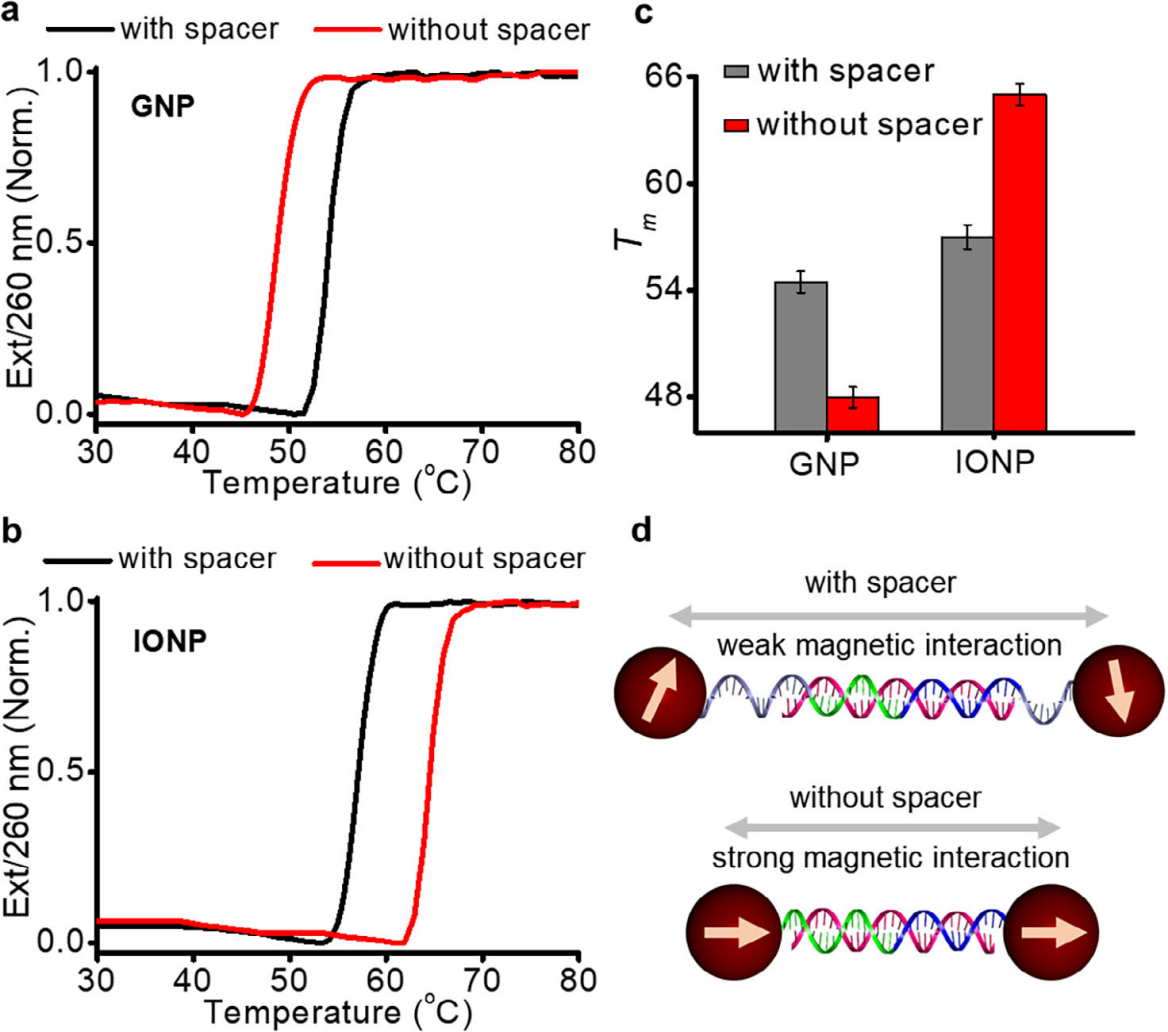
The effect of NP type on DNA denaturation properties. a) Melting profiles of GNP1S/GNP2S and GNP1/GNP2. b) Melting profiles of IONP1S/IONP2S and IONP1/IONP2. c) *T_m_* of IONP and GNP assemblies with and without A_10_ spacers. d) Scheme illustrating the length‐dependent magnetic interaction.

To investigate the effect of NP type on DNA interactions, pure IONP (17 nm) and GNP (12 nm) assemblies were prepared using four different pairs of building blocks: IONP1/IONP2, IONP1S/IONP2S, GNP1/GNP2, and GNP1S/GNP2S (**Figure** **4**). All four combinations exhibited DNA‐induced NP aggregation upon adding the linker DNA, L21 (Figure S12, Supporting Information). DNA denaturation analyses of GNP assemblies (GNP1/GNP2 and GNP1S/GNP2S) showed that the incorporation of additional A_10_ spacer increased *T*_m_ by ≈6 °C (Figure 4a; Figure S13a, Supporting Information). This spacer effect for GNPs has been attributed to the enhanced DNA binding due to reduced steric hindrance.^[^ ^36^ ^]^ Interestingly, IONP assemblies demonstrated the opposite effect (Figure 4b; Figure S13b, Supporting Information), where IONP1/IONP2 assemblies exhibited an 8 °C higher *T*_m_ compared to IONP1S/IONP2S assemblies. This distinct spacer effect is clearly illustrated in Figure 4c. The reduced *T*_m_ with the spacer is attributed to the increased interparticle distance which reduces the magnetic dipole interaction between IONPs (Figure 4d). The small angle x‐ray scattering (SAXS) analyses (Figure S14a, Supporting Information) confirmed the increase in the center‐to‐center interparticle distance from 22.0 to 35.3 nm with the incorporation of the spacer. The magnetic dipole interaction energy (*U*_dd_)^[^ ^37^, ^38^ ^]^ was calculated to be ‐5.5 *kT* for an IONP (17 nm) dimer separated by 22.0 nm (see Supporting Information for details). This value is comparable to the dinucleotide interaction energy (≈‐2.4 *kT*),^[^ ^39^ ^]^ indicating that the magnetic interaction is substantial enough to influence DNA‐mediated assembly at short interparticle distances. The magnetic interaction in an IONP dimer separated by 35.3 nm was calculated to be ‐1.3 *kT* (close to 1 *kT*, thermal energy at room temperature), indicating that the magnetic contribution is weak in the IONP pair. This finding is consistent with a previous study showing enhanced supramolecular bonding in polymer‐coated IONPs due to magnetic interactions.^[^ ^40^, ^41^ ^]^

### 2.4 Length‐Dependent Magnetic Reinforcement

**Figure 5:**
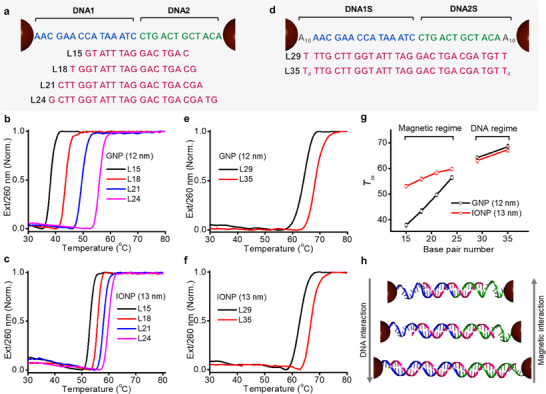
DNA length dependence. a) DNA sequences used for the thermal denaturation experiments carried out with L15‐L24. b,c) Melting profiles of 12 nm GNP (b) and 13 nm IONP (c) assemblies formed with L15‐L24 DNA linkers. d) DNA sequences used for the thermal denaturation experiments carried out with L29‐L35. e,f) Melting profiles of 12 nm GNP (e) and 13 nm IONP (f) assemblies formed with L29‐L35 DNA linkers. g) *T*_m_ of GNP and IONP assemblies formed with L15‐L35 DNA linkers. h) Illustration of the impact of linker lengths on magnetic and DNA interactions.

Since both DNA and magnetic interactions collectively influence the thermal stability of NP assemblies, the well‐known principle of DNA hybridization may not fully apply to DNA‐linked IONP assemblies. To explore this further, we investigated the effect of DNA linker lengths on the thermal stability of DNA‐linked NPs (**Figure** **5**). In this set of experiments, GNP (12 nm) and IONP (13 nm) assemblies were constructed using a series of DNA linkers with varying lengths (15 to 24 bases), labeled as L15, L18, L21, and L24, respectively (Figure 5a; Table S1, Supporting Information). In this design, the interparticle distance increases with the length of the linker, as double‐stranded DNA (dsDNA) is more rigid than single‐stranded DNA (ssDNA).^[^ ^42^, ^43^, ^44^ ^]^ The gradual increase in the interparticle distance with the linker length was confirmed by SAXS measurements (Figure S14, Supporting Information).

The thermal denaturation analysis of GNP assemblies demonstrated a significant increase in *T_m_* with increasing DNA linker lengths (Figure 5b; Figure S15a, Supporting Information). On the contrary, the *T_m_* of IONP assemblies exhibited only slight increases with increasing linker lengths (Figure 5c; Figure S15b, Supporting Information). For IONPs, the thermal stabilization gained by increasing the number of DNA base pairs is counterbalanced by the destabilization caused by the reduced magnetic interaction (Figure 5c). In general, IONP assemblies showed higher *T_m_* than GNP assemblies, attributed to the additional stabilization provided by the magnetic dipole interaction (Figure 5b,c). The difference in *T_m_* between IONP and GNP decreases with increasing linker lengths due to the reduction in magnetic contribution at longer interparticle distances. Additional experiments with 17 nm IONPs showed greater increment in *T*_m_ than 13 nm IONPs due to greater magnetic interactions between larger particles (Figure S16, Supporting Information).

We anticipated that IONPs may exhibit *T_m_* values similar to those of GNPs, as the magnetic interaction becomes negligible with a further increase in the linker length. Indeed, GNP1S/GNP2S and IONP1S/IONP2S assemblies prepared with L29 and L35 linkers (Figure 5d) showed similar *T*_m_ values (Figure 5e,f; Figure S15c,d, Supporting Information), indicating that the DNA interaction is dominant in this regime. Overall, the comparison between GNP and IONP data indicates that the length dependence of *T*_m_ in IONPs can be divided into two regimes, as illustrated in Figure 5g,h: 1) the magnetic‐dominant regime at short interparticle distances, where both magnetic and DNA interactions affect *T_m_*, and 2) the DNA‐dominant regime at long interparticle distances, where *T_m_* is predominantly determined by DNA interactions. In the magnetic‐dominant regime, the magnetic interaction can enhance the thermal stability of IONP assemblies at short interparticle distances, leading to unusually weak DNA length dependence of *T*_m_ (Figure 5h). The transition point between the magnetic and DNA regime is expected to shift to a longer interparticle distance with increasing IONP size, with a threshold NP size above which magnetic interactions become significant.^[^ ^38^ ^]^

## 3 Conclusion

In summary, we developed a straightforward DNA functionalization method for IONPs using GA‐DNA, which is as simple as the DNA functionalization method for GNPs using thiolated DNA. Note that the synthesis of GA‐DNA can be carried out by the automated solid‐state synthesis through the incorporation of galloylated phosphoramidite. GA‐DNA efficiently binds to the IONP surface through chelated metal coordination, demonstrating stability across various buffer conditions. The synthetic approach involving a direct DNA attachment without the need for a polymer or silica interlayer allowed for in‐depth investigation of unique molecular recognition properties of IONP‐DNA. In general, DNA‐linked IONPs showed higher *T_m_* than DNA‐linked GNPs, indicating that the magnetic dipole interactions can enhance the thermal stability of DNA‐linked IONPs. This magnetic enhancement is more pronounced at shorter interparticle distances, resulting in an unusually weak dependence of *T_m_* on DNA linker lengths. The magnetic effect diminishes at sufficiently long interparticle distances. Overall, the behavior of DNA‐functionalized IONPs can be classified into two regimes: a magnetic‐dominant regime and a DNA‐dominant regime, both of which should be considered when designing DNA sequences for self‐assembly of superparamagnetic NPs. The simple DNA functionalization method presented here can be applied to other metal compound NPs exhibiting strong affinity to the gallol group. Furthermore, the findings in this study highlight the exciting possibility of using magnetic interactions to control biomolecular interactions.

## 4 Experimental Section

### Synthesis of IONP‐DNA

IONPs of 13.3 and 17.1 nm were synthesized according to a previously reported protocol.^[^ ^32^ ^]^ HPLC‐purified GA‐DNA was desalted by 10 k molecular weight cut off filters (ultrafiltration at 7000 rpm for 4 min, 10 times) prior to use. Direct functionalization of DNA to IONPs was achieved by mixing IONPs (20 nM, 1 eq. 13 or 17 nm) with GA‐DNA (0.010 mM, 500 eq., for 13 nm IONPs, and 0.015 mM, 750 eq., for 17 nm IONPs) in 1 mL of 80% THF in water. The mixture was incubated at room temperature on a shaker at 500 rpm for 5 h. (Note: In the 80% THF solution, IONPs remained well‐dispersed in the presence of desalted GA‐DNA. However, without the desalting step, IONPs settled within 10 min. In both cases, IONPs became well‐dispersed upon increasing the water content to 50%). Subsequently, the water ratio was increased to 50%, followed by further incubation for 12 h. The THF was then evaporated under a nitrogen stream. The solution was adjusted to 0.1 m PBS by slowly adding 1 M PBS over 60 min, followed by a 12 h incubation. The reaction vial was then immersed in liquid nitrogen for 2 min, slowly brought to room temperature, and this freeze‐thaw cycle was repeated five times to obtain stable IONP‐DNA conjugates. The conjugates were purified by centrifugation (15,000 rpm, 10 min, 10 °C), and the final IONP‐DNA conjugates were dispersed in 0.3 m PBS.

### Thermal Denaturation Studies of DNA‐Linked IONP and GNP

The self‐assembly of IONP/IONP, IONP/GNP, and GNP/GNP was induced by adding appropriate linkers to corresponding NP mixtures. In detail, a PBS solution (1 mL, 0.3 m, pH 7.4) containing IONP‐DNA or GNP‐DNA (2.5 nm, 1 eq.), counter IONP‐DNA or GNP‐DNA (2.5 nm, 1 eq.), and a suitable linker (250 nm, 100 eq.) was heated at 60 °C for 5 min, followed by incubation at room temperature for 3 h. Before conducting thermal denaturation experiments, the solution was preheated at 10 °C below the *T*_m_ for 10 min. Thermal denaturation experiments were carried out by collecting UV–vis spectra while heating the NP aggregate solution from 25 to 80 °C, with a 1 °C increase per min and a 1 min holding time at each temperature.

### Quantification of DNA on IONP

IONPs (0.8 nM, 13 nm) were conjugated with GA‐DNA (400 nM, DNA 1) using the method described above. The solution was centrifuged at 15,000 rpm for 20 min at 10 °C to remove unconjugated GA‐DNA. The concentration of unreacted DNA in the supernatant was determined using UV–vis absorption spectroscopy. The amount of conjugated DNA was calculated by subtracting the amount of unreacted DNA from the initial amount of DNA. The number of DNA strands attached to each IONP was then calculated by dividing the concentration of conjugated DNA by the concentration of IONPs determined by the ICP analysis.

### Determination of Interparticle Distance

Interparticle distances in DNA‐linked IONP assemblies were determined by synchrotron SAXS analyses. For 17 nm IONPs, IONP1/IONP2 and IONP1S/IONP2S assemblies were prepared with L21 in 0.3 M PBS. For 13 nm IONPs, IONP1/IONP2 assemblies were prepared with L15, L18, L21, or L24, while IONP1S/IONP2S assemblies were prepared with L29 or L35. The IONP and DNA linker concentrations were kept constant at 2.5 and 250 nm, respectively for all samples. SAXS measurements were also conducted for 0.3 m PBS and dispersed IONP‐DNA (2.5 nm) to correct for the background signal and determine the form factor, respectively. To obtain accurate scattering intensities, *I* (*q*), the background signal from a capillary containing 0.3 m PBS buffer was subtracted from each data. Finally, the interparticle distance was calculated from the form factor and pair distribution function (PDF) analysis. The structure factor, *S*(*q*), of IONP assemblies was obtained from the background‐corrected scattering intensity, *I*_Sub_ (*q*) of DNA‐linked IONPs using a spherical form factor and a hard sphere potential model with the SasView software.

## Conflict of Interest

The authors declare no conflict of interest.

## Supporting information



Supporting Information

## Data Availability

The data that support the findings of this study are available from the corresponding author upon reasonable request.
