# Supplementary material for: Iron Oxide Nanoparticles Modified with Galloylated DNA for Magnetically Enhanced DNA‐Directed Assembly
Source: Adv Sci (Weinh). 2025 Jun 29;12(32):e01491. doi: 10.1002/advs.202501491 (PMC12407300; doi:10.1002/advs.202501491)
Supplement: Supplementary file 1 — Supporting Information [file ADVS-12-e01491-s001.docx]

Supporting Information

**Iron Oxide Nanoparticles Modified with Galloylated DNA for Magnetically Enhanced DNA-Directed Assembly**

*Murali Golla, Hyunjin Jeon, Shine K. Albert, Hojun Lee, Seulki Kang, Moon Jeong Park and So-Jung Park**

M. Golla, H. Jeon, S. K. Albert, S. Kang, S.-J. Park

Department of Chemistry and Nanoscience, Ewha Womans University, Seoul, 03760, Republic of Korea

H. Lee, M. J. Park

Department of Chemistry, Pohang University of Science and Technology (POSTECH), Pohang, 37673, Republic of Korea

M. Golla, S.-J. Park

Graduate Program in Innovative Biomaterials Convergence, Ewha Womans University, Seoul, 03760, Republic of Korea

E-mail: sojungpark@ewha.ac.kr (So-Jung Park)

**I. Detailed procedures**

**Materials and instrumentations.** All chemicals used for organic syntheses were purchased from Sigma-Aldrich and TCI and organic solvents were dried following standard protocols. Oligonucleotides were purchased from Bioneer (Daejeon, Korea). Gel electrophoresis reagents were purchased from Bio-Rad and used without additional treatment. Ultrapure water (18 MΩ, Millipore) was used in all experiments. Thin-layer chromatography (TLC) analyses were performed on aluminum plates coated with silica gel 60 F254. Column chromatography was conducted using silica gel 60 with a particle size of 0.063–0.2 mm. ^1^H and ^13^C nuclear magnetic resonance (NMR) spectra were recorded on a 300 MHz Bruker Avance spectrometer, with tetramethylsilane (TMS) as the internal standard. Galloylated DNA (GA-DNA) was purified by high-performance liquid chromatography (HPLC) equipped with a UV detector (YL Instruments Co., Ltd., Gyeonggi-do, Korea). HPLC analysis was performed on a C5 column (5 μm particle size, 250 × 10 mm, 100 Å pore size, Phenomenex, CA, USA) using gradient elution with 20 mM ammonium acetate and acetonitrile. The flow rate was set to 2 mL/min, and detection was carried out at a wavelength of 254 nm. The molecular weights of GA-DNA were determined using matrix-assisted laser desorption/ionization time-of-flight (MALDI-TOF) mass spectrometry, with assistance from Bioneer. Extinction spectra and melting transition studies were conducted using a UV-Vis spectrophotometer equipped with a temperature controller (Agilent Technologies). Dynamic light scattering (DLS) measurements were performed using a Zetasizer Nano-ZS (Malvern Instruments, Malvern, UK) operating with a 632.8 nm laser at a scattering angle of 173^o^. Transmission electron microscopy (TEM) images were acquired using a JEM-2100F microscope (JEOL). TEM samples were prepared by depositing 5 μL of the sample onto a 400-mesh carbon-coated copper grid (Ted Pella, Inc.), allowing it to be adsorbed for 2 min, wicking off the excess liquid with a filter paper and the grids were allowed to be air-dried. Synchrotron small-angle X-ray scattering (SAXS) measurements were conducted at the Pohang Accelerator Laboratory (PAL) on the PLS-II 9A beamline, equipped with a two-dimensional detector. Samples were loaded into quartz glass capillaries (Capillary Tube Supplies Ltd, QGCT 1.5) and sealed with Teflon tops. The incident X-ray beam had a wavelength (λ) of 0.063 nm (Δλ/λ = 10⁻⁴), and the sample-to-detector distance was set at 4.5 m.

**Table S1.** DNA sequences.

| **Name** | **Sequence (5’ to 3’)** |
| --- | --- |
| DNA1S | CTA AAT ACC AAG CAA-A_10_-**GA** |
| DNA2S | **GA**-A_10_-ACA TCG TCA GTC-3 |
| DNA1 | CTA AAT ACC AAG CA-**GA** |
| DNA2 | **GA**-ACA TCG TCA GTC |
| DNA1S-SH | CTA AAT ACC AAG CAA-A_10_-**SH** |
| DNA2S-SH | **SH**-A_10_-ACA TCG TCA GTC |
| DNA1-SH | CTA AAT ACC AAG CA-**SH** |
| DNA2-SH | **SH**-ACA TCG TCA GTC |
| L15 | GTA TTT AGG ACT GAC |
| L18 | TGG TAT TTA GGA CTG ACG |
| L21 | CTT GGT ATT TAG GAC TGA CGA |
| L24 | GCT TGG TAT TTA GGA CTG ACG ATG |
| L29 | TTT GCT TGG TAT TTA GGA CTG ACG ATG TT |
| L35 | TTT TTT GCT TGG TAT TTA GGA CTG ACG ATG TTT TT |

**
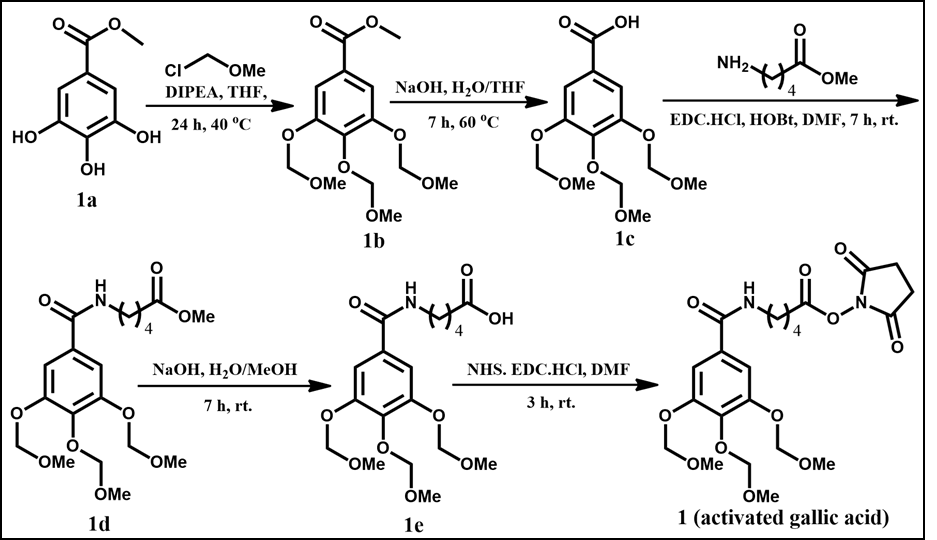
Synthesis and characterization of activated gallic acid (1).** The activated gallic acid was synthesized following the multistep organic synthesis outlined in Scheme S1.^[1]^

**Scheme S1.** Scheme showing the synthesis of activated gallic acid (**1**)**.**


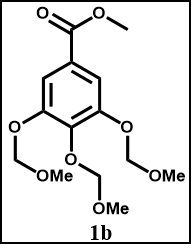
**Synthesis of compound 1b.** Methyl 3,4,5-trihydroxybenzoate (2 g, 10.84 mmol) was dissolved in 30 mL anhydrous tetrahydrofuran (THF). To this solution, DIPEA (7.0 mL) and chloromethoxy methyl chloride (7.8 g, 97.56 mmol) were added at 0 °C. The reaction mixture was then allowed to warm to room temperature and stirred at 50 °C for 40 h. After completion, the crude product was extracted with ethyl acetate and purified by silica column chromatography (neutralized with triethylamine) using an n-hexane:ethyl acetate (20:80) solvent system to yield the pure product (Yield: 72.9%). TLC analysis (n-hexane:ethyl acetate 20:80) showed an *R*_f_ value of 0.37. ^1^H NMR (300 MHz, DMSO) δ (ppm): 7.41 (s, 2H), 5.26 (s, 4H), 5.14 (s, 2H), 3.83 (s, 3H), 3.51 (s, 3H), 3.41 (s, 6H). ^13^C NMR (125 MHz, DMSO) δ (ppm): 166.05, 150.95, 140.56, 125.41, 110.85, 98.20, 95.13, 57.02, 56.33, 52.75.

**Synthesis of compound 1c.** A solution of NaOH (0.68 g, 17.08 mmol) in 20 mL H_2_O was added to a solution of compound **1b** in 20 mL methanol at 0 °C. The reaction mixture was stirred at 60 °C for 4 h. After the reaction, the crude product was carefully extracted using ethyl acetate and dilute HCl (0.5). The solvent was evaporated under reduced pressure, and the
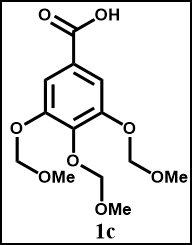
obtained pure product was used directly in the next step without further purification (Yield: 81.2%). ^1^H NMR (300 MHz, DMSO) δ (ppm): 7.40 (s, 2H), 5.24 (s, 4H), 5.13 (s, 2H), 3.51 (s, 3H), 3.41 (s, 6H). ^13^C NMR (125 MHz, DMSO) δ (ppm): 167.13, 150.82, 140.24, 126.68, 111.08, 98.19, 95.13, 56.99, 56.30.


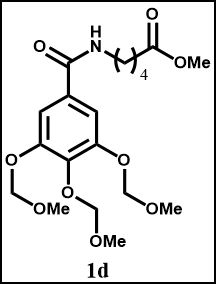
**Synthesis of compound 1d.** A mixture of compound **1c** (0.4 g, 1.32 mmol) and methyl 6-aminohexanoate (0.36 g, 1.98 mmol) was dissolved in 10 mL anhydrous dimethylformamide (DMF). To this solution, EDC·HCl (0.30 g, 1.98 mmol), HOBt (0.26 g, 1.98 mmol), and DMAP (0.03 g, 0.26 mmol) were added under nitrogen at 0 °C. The reaction mixture was then allowed to warm to room temperature and stirred for 7 h. The product was extracted with ethyl acetate, and the solvent was removed under reduced pressure. The crude reaction mixture was purified by silica column chromatography using ethyl acetate (column neutralized with triethylamine) to obtain the desired product (Yield: 80.1 %). TLC analysis (ethyl acetate) showed an *R*_f_ value of 0.39. ^1^H NMR (300 MHz, DMSO) δ (ppm): 8.38 (t, *J* = 6 Hz, 1H), 7.29 (s, 2H), 5.24 (s, 4H), 5.10 (s, 2H), 3.58 (s, 3H), 3.51 (s, 3H), 3.41 (s, 6H), 3.21 (m, 2H), 2.31 (t, *J* = 6 Hz, 2H), 1.50 (m, 4H), 1.30 (m, 2H). ^13^C NMR (125 MHz, DMSO) δ (ppm): 173.80, 165.72, 150.61, 150.60, 138.87, 130.67, 109.31, 98.18, 95.12, 56.97, 56.36, 51.64, 33.66, 29.30, 26.41, 24.67.


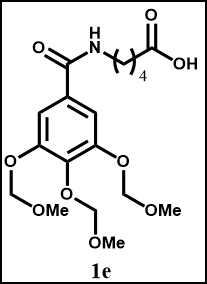
**Synthesis of compound 1e.** Compound **1d** was dissolved in 6 mL of a 1:1 mixture of methanol and THF. To this, a solution of NaOH in 6 mL of water was added, and the reaction mixture was stirred at room temperature overnight. After the reaction, the compound was carefully extracted using ethyl acetate and dilute HCl, and the solvent was evaporated under reduced pressure. The obtained pure product was used directly in the next step without further purification. (Yield: 84.3 % ) ^1^H NMR (300 MHz, DMSO) δ (ppm): 8.37 (t, *J* = 6 Hz, 1H), 7.28 (s, 2H), 5.24 (s, 4H), 5.10 (s, 2H), 3.51 (s, 3H), 3.42 (s, 6H), 3.21 (m, 2H), 2.21 (t, *J* = 6 Hz, 2H), 1.50 (m, 4H), 1.33 (m, 2H). ^13^C NMR (125 MHz, DMSO) δ (ppm): 174.93, 165.70, 150.60, 138.86, 130.68, 109.29, 98.18, 95.12, 56.97, 56.37, 34.06, 29.38, 26.52, 24.73.

**Synthesis of activated gallic acid (1).** A mixture of compound **1e** (0.13 g, 0.13 mmol) and N-hydroxysuccinimide (0.04 g, 0.40 mmol) was dissolved in 5 mL of anhydrous DMF. To this solution, EDC·HCl (0.06 g, 0.40 mmol) was added under nitrogen at 0 °C. The reaction mixture was then allowed to warm to room temperature and stirred for 3 h. The product was extracted with ethyl acetate, and the solvent was removed under reduced pressure. The crude reaction mixture was purified by silica column chromatography using ethyl acetate (filtration column) to obtain the desired product (Yield: 79.6%). The total yield for the multistep synthesis of **1** was
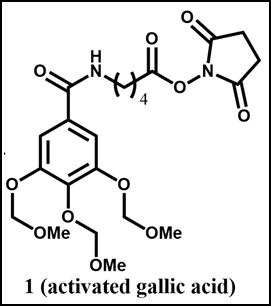
determined to be 37.7%. TLC analysis (ethyl acetate) showed an *R*_f_ value of 0.4. ^1^H NMR (300 MHz, DMSO) δ (ppm): 8.38 (t, *J* = 6 Hz, 1H), 7.28 (s, 2H), 5.24 (s, 4H), 5.10 (s, 2H), 3.50 (s, 3H), 3.41 (s, 6H), 3.20 (m, 2H), 2.81 (s, 4H), 2.68 (t, *J* = 6 Hz, 2H), 1.68 (m, 2H), 1.61 (m, 2H), 1.54 (m, 2H). ^13^C NMR (125 MHz, DMSO) δ (ppm): 174.93, 165.70, 150.60, 138.86, 130.68, 109.29, 98.18, 95.12, 56.97, 56.37, 34.06, 29.38, 26.52, 24.73.

**
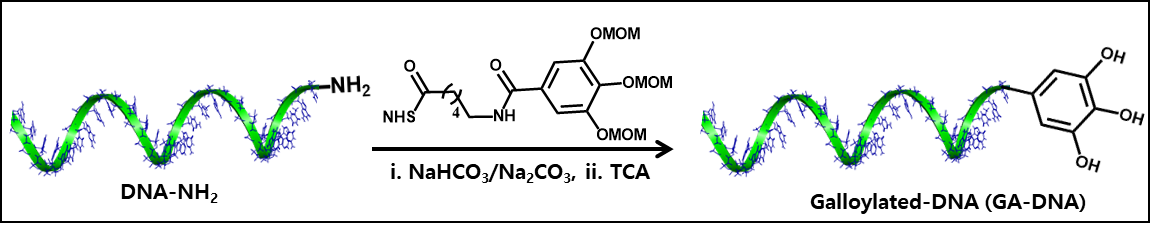
Synthesis of GA-DNA.** GA-DNA was synthesized by reacting activated gallic acid with amine-modified DNA under the basic condition.^[2]^ Amine-modified DNAs (170 nmol, DNA1S/DNA2S/DNA1/DNA2) were dissolved in sodium carbonate/bicarbonate buffer (5 mM, 0.2 mL) and incubated at room temperature for 30 min. The solution was added to activated gallic acid (8.7 mg, 17,000 nmol) in 0.5 mL of DMF. The DNA vial was rinsed with 0.3 mL of water, and the rinse solution was added to the reaction mixture. The reaction was incubated at room temperature for 24 h with vigorous stirring. After the reaction, an ethyl acetate and water workup was performed to remove excess activated gallic acid and DMF. The crude reaction mixture was purified by HPLC using a C5 reverse-phase column with gradient elution of ammonium acetate and acetonitrile. The HPLC-purified DNA fractions were combined, and excess solvent was removed under reduced pressure, with the final volume adjusted to 1 mL. The HPLC-purified DNAs were treated with 0.5 mL of 3% TCA for 1 h, after which the TCA was removed under reduced pressure. The DNAs were then passed through desalting columns to obtain pure GA-DNA. The yield of the DNA coupling reaction ranged from 72.3% to 85.0%.

**Scheme S2.** Illustration of the synthesis of GA-DNA.

**Synthesis of DNA-grafted gold nanoparticles (GNP-DNA).** GNP-DNA conjugates were synthesized using a previously reported salt-aging method.^[3]^ A solution containing 12.1 nm GNPs (17 nM) and thiol-modified DNAs (3.16 µM, DNA1S-SH, DNA2S-SH, DNA1-SH or DNA2-SH) in 5 mL of water was incubated on an orbital shaker for 16 h. The buffer concentration was then adjusted to 0.1 M by adding 1 M PBS over 3 h. The solution was further incubated on an orbital shaker for 40 h at room temperature. Excess thiolated DNAs were removed by centrifugation at 12,000 rpm for 30 min, after which the particles were dispersed in 0.3 M PBS. The DNA-functionalized GNPs were characterized using UV-Vis absorption spectroscopy and zeta potential analysis.

**Calculation of magnetic interaction energy of IONP dimers.** The magnetic dipole-dipole interaction energy (*U*_dd_) can be estimated using Equation S1,^[4-6]^ where *m* is the magnetic moment, *μ_0_* is the magnetic permeability, *d* is the center-to-center interparticle distance, *V* is the NP volume, and *M*_s_ is the saturation magnetization (4.19 × 10^5^ A/m for 17 nm IONP and 3.93 × 10^5^ A/m for 13 nm IONP).^[7-8]^

*U* _dd_ $\mathbf{=}\frac{{-m}^{2}}{2\pi{\mu_{0}d}^{3}}$

$\mathbf{=}\frac{{-(\mu_{0}M_{s}V)}^{2}}{2\pi{\mu_{0}d}^{3}}$ (Equation S1)

The *U*_dd_ was calculated to be -5.5 *kT* and -1.3 *kT* for a dimer of 17.1 nm IONP separated by center-to-center interparticle distances of 22.0 nm (corresponding to IONP1/IONP2 linked with L21) and 35.3 nm (IONP1S/IONP2S linked with L21), respectively. The calculated *U*_dd_ values for 13.3 nm IONP dimers formed with various linkers are listed in Table S2, showing the reduction in the magnetic energy with increasing the interparticle distance.

**Table S2.** Calculated *U*_dd_ of 13.3 nm IONP dimers with various linkers.

| Linker^1^ | L15 | L18 | L21 | L24 | L29 | L35 |
| --- | --- | --- | --- | --- | --- | --- |
| Interparticle distance from SAXS data (nm) | 16.8 | 17.7 | 19.3 | 19.9 | 24.0 | 25.6 |
| *U_dd_* (*kT*) | -2.40 | -2.06 | -1.58 | -1.45 | -0.82 | -0.68 |

^1^Linkers L15, L18, L21, and L24 were used to assemble IONP1 and IONP2, and linkers L29 and L35 were used to assemble IONPS1 and IONPS2.

**II. Supporting data**


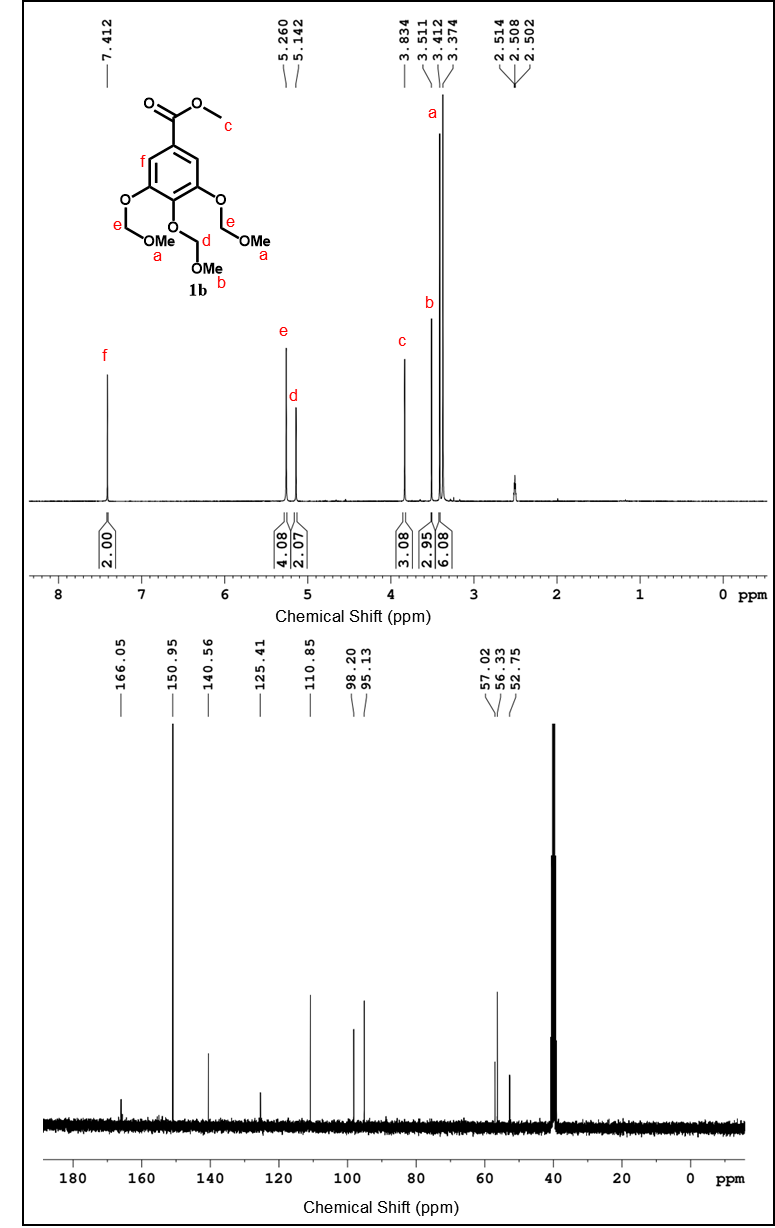


**Figure S1.** ^1^H (top) and ^13^C (bottom) NMR spectra of **1b.**


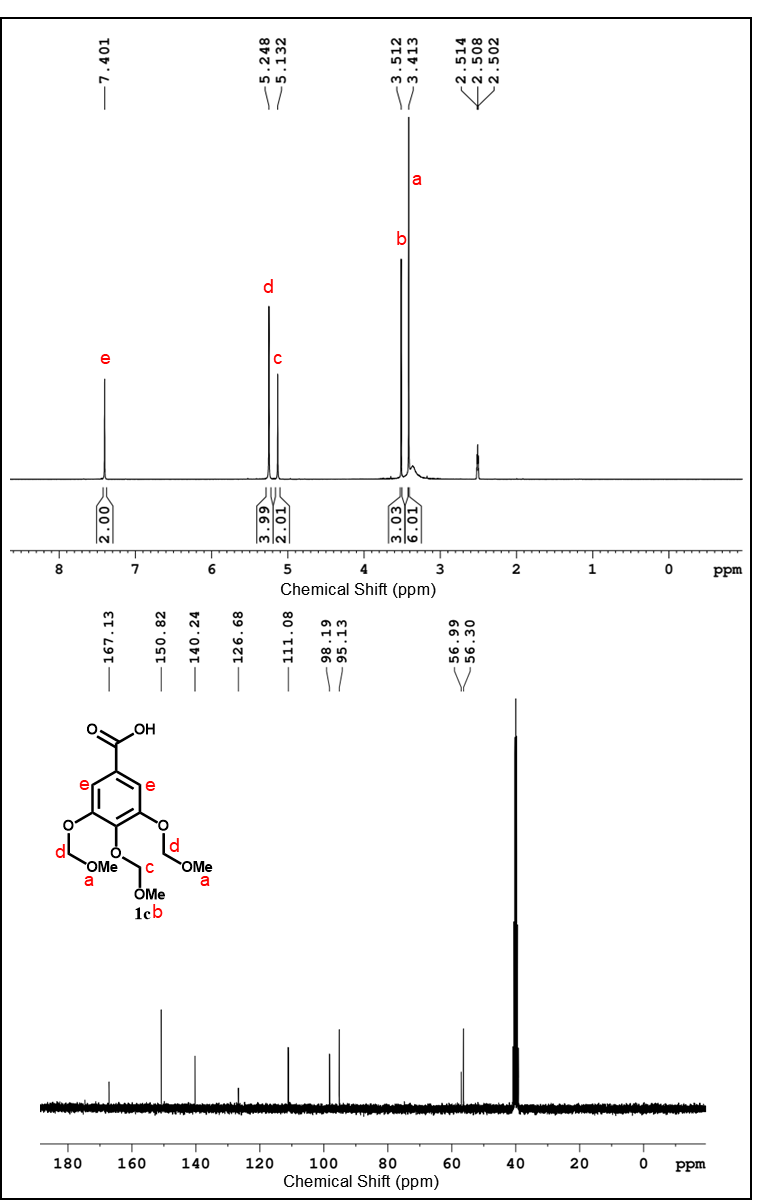


**Figure S2.** ^1^H (top) and ^13^C (bottom) NMR spectra of **1c.**


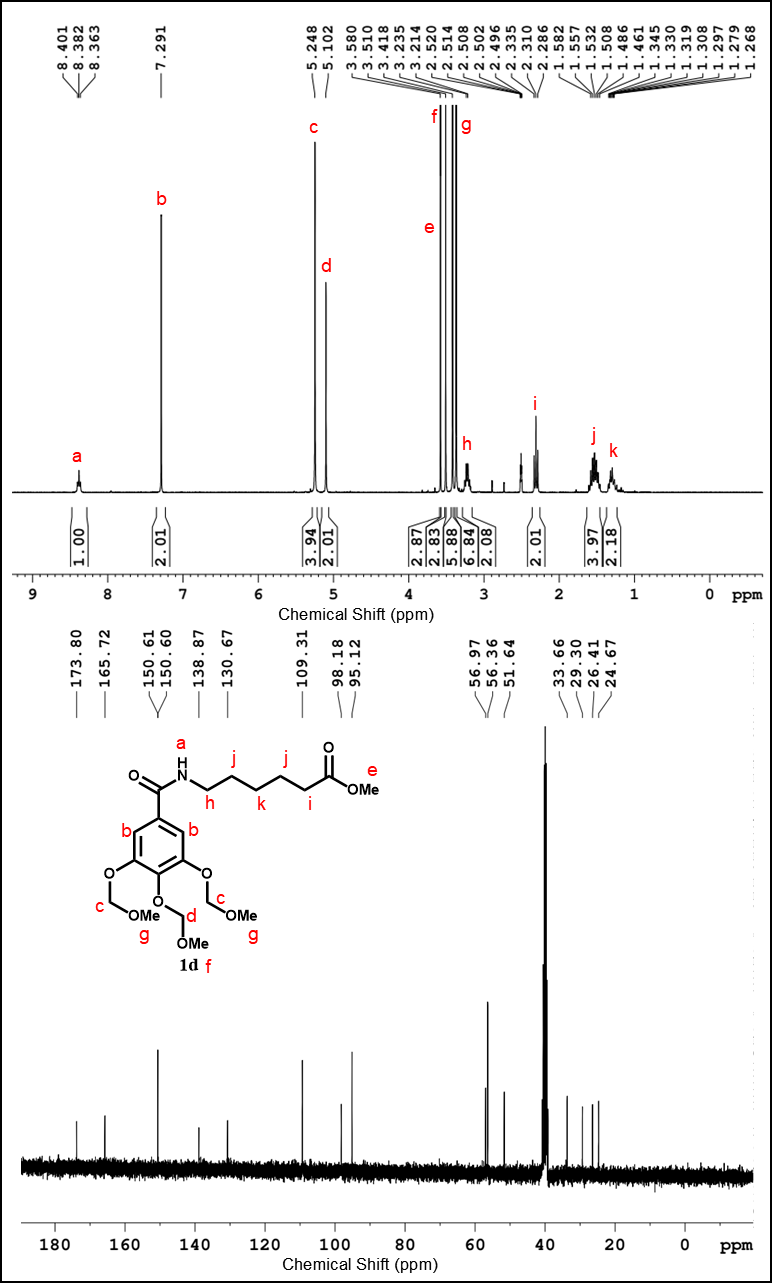


**Figure S3.** ^1^H (top) and ^13^C (bottom) NMR spectra of **1d.**

**
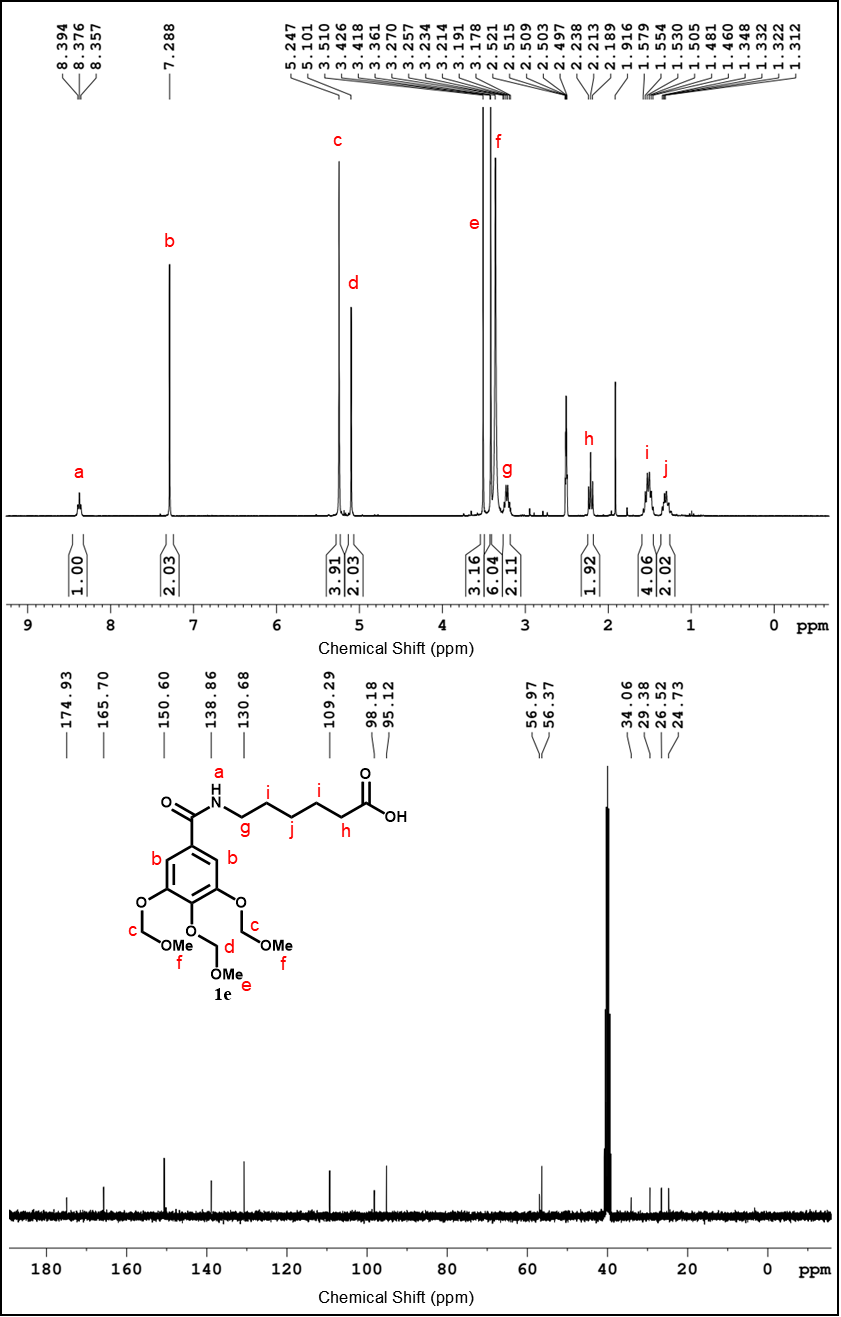
**

**Figure S4.** ^1^H (top) and ^13^C (bottom) NMR spectra of **1e.**


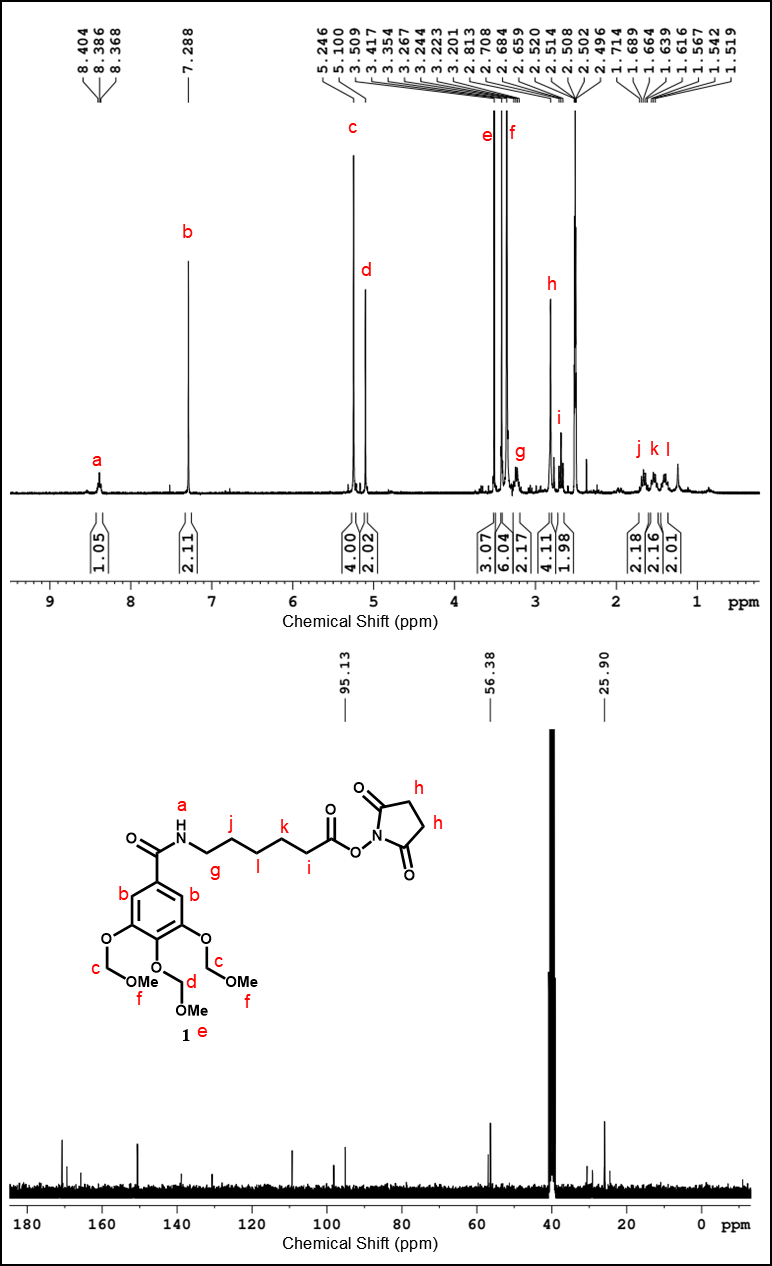


**Figure S5.** ^1^H (top) and ^13^C (bottom) NMR spectra of **1.**

**
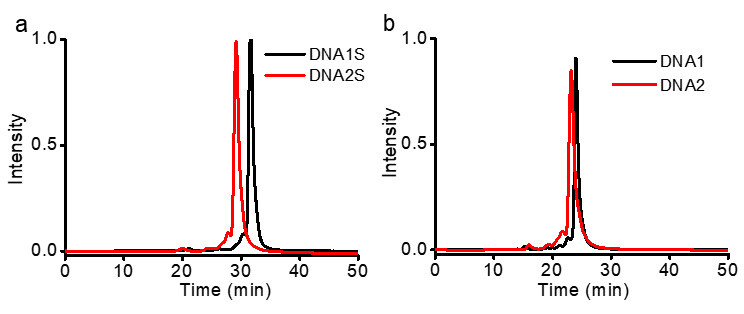
**

**Figure S6.** Analytical HPLC profiles of GA-DNAs. (a) DNA strands with spacers. (b) DNA strands without spacers. The black and red traces in both spectra represent the 3' and 5' modified GA-DNAs, respectively.

**
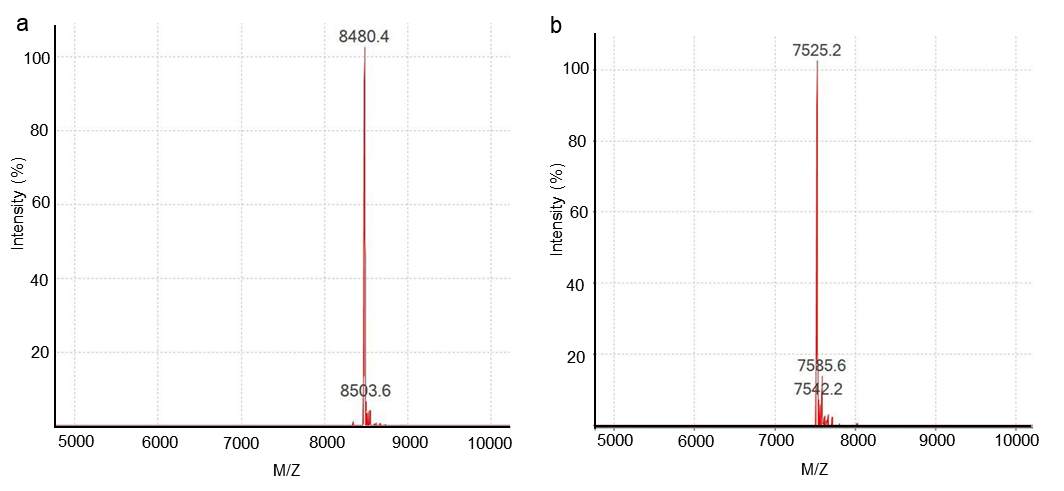
**

**Figure S7.** MALDI-TOF spectra of (a) DNA1S and (b) DNA2S.

**Figure S8.** UV-Vis absorption spectra used to calculate the number of DNA strands per IONP.


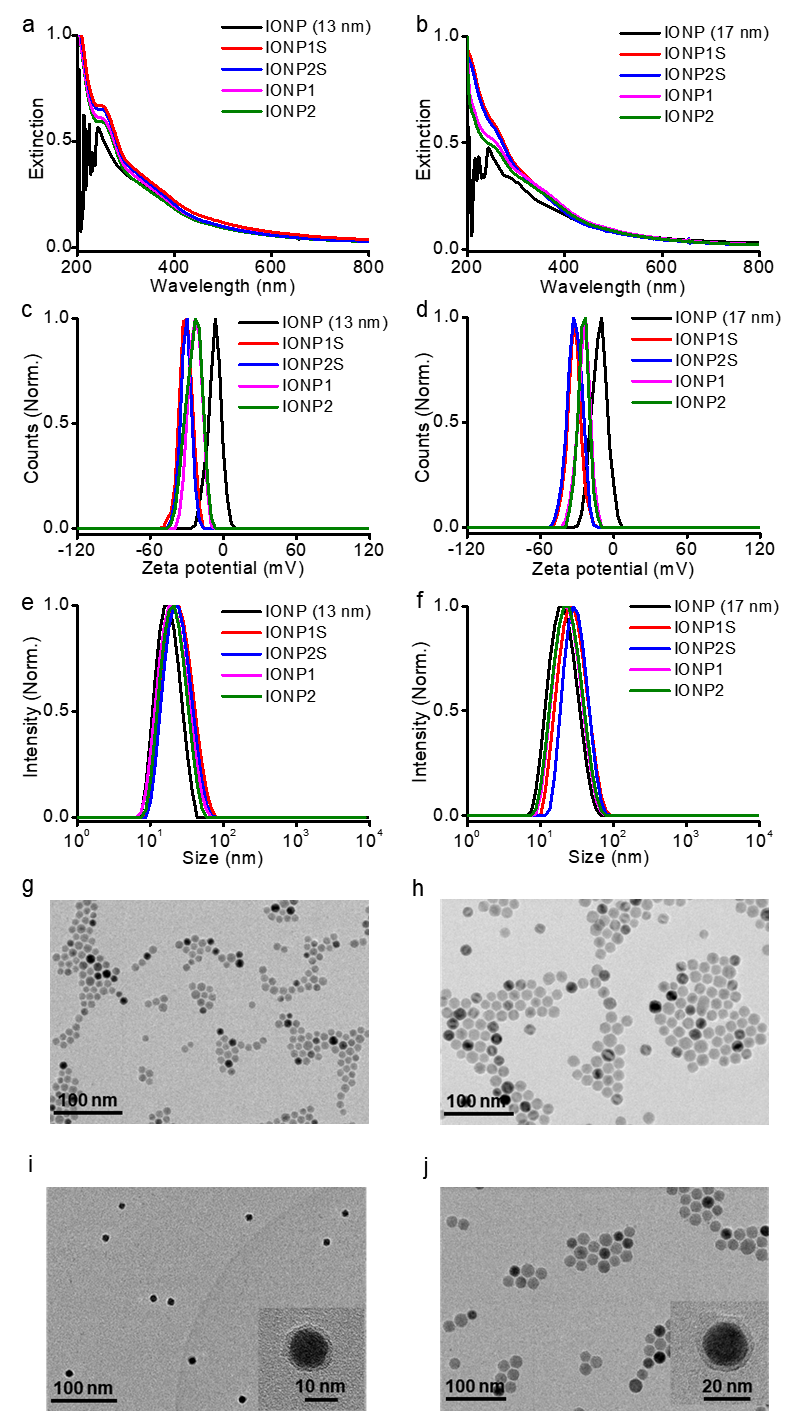


**Figure S9.** Spectroscopic and morphological characterizations of IONP and IONP-DNA. (a) Extinction spectra of 13 nm IONP before and after DNA modification. (b) Extinction spectra of 17 nm IONP before and after DNA modification. (c) Zeta potential trace of 13 nm IONP before and after DNA modification. (d) Zeta potential trace of 17 nm IONP before and after DNA modification. (e) DLS profiles of 13 nm IONP and IONP-DNA. (f) DLS profiles of 17 nm IONP before and after DNA modification. (g-h) TEM images of as-synthesized 13 nm IONPs (g) and 17 nm IONPs (h). (i-j) TEM images of 13 nm IONP1S (j) and 17 nm IONP1S (j). Insets in (i) and (j) show zoomed-in images of IONP1S stained with uranyl acetate, highlighting a DNA shell around the particle.


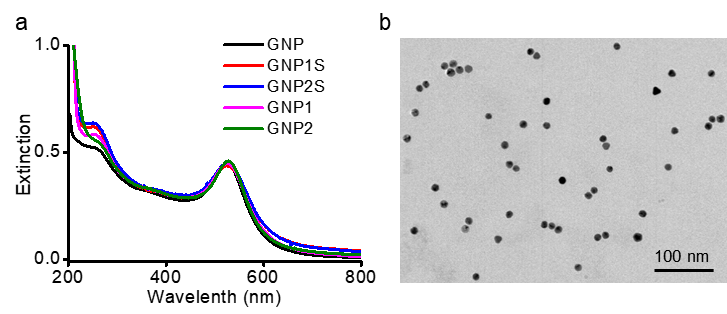
**
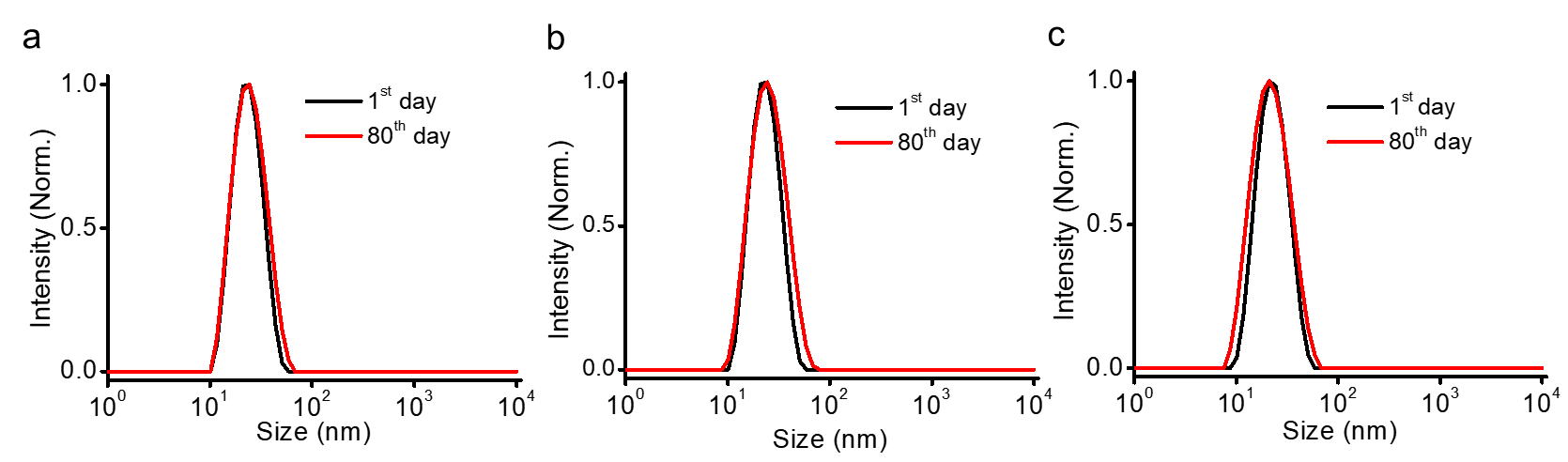
Figure S10.** Colloidal stability of DNA-modified IONP. (a–c) DLS data of 13 nm IONP1S dispersed in 0.3 M TBE (a), PBS (b), and Tris (c) buffers, measured on the 1st and 80^th^ days after synthesis.


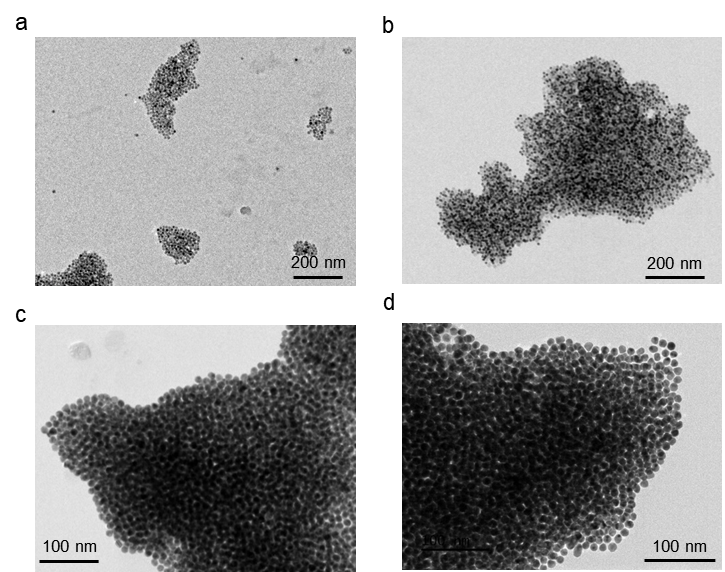
**Figure S11.** Characterizations of GNP-DNA. (a) Extinction spectra of GNP before and after DNA-functionalization. (b) TEM image of GNP1S.

**Figure S12.** TEM images of self-assembled NPs in the presence of linker L21. (a) IONP1S/IONP2S. (b) IONP1/IONP2. (c) GNP1S/GNP2S. (d) GNP1/GNP2.

**
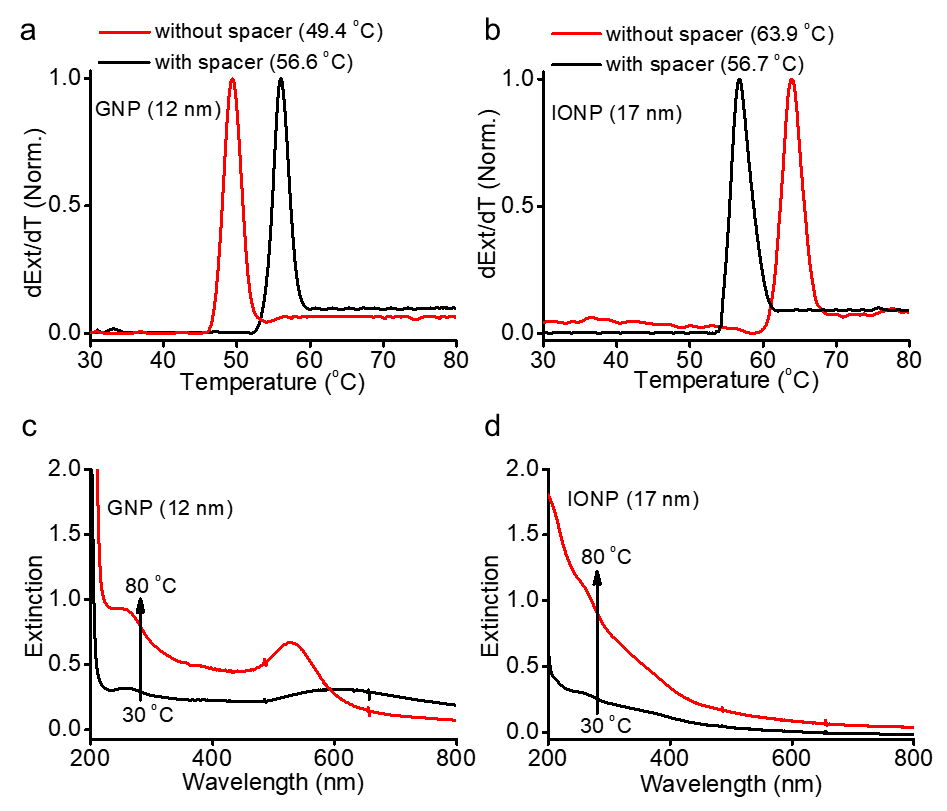
Figure S13.** Thermal denaturation data of L21-linked NP assemblies. (a-b) Derivative plots of (a) GNP assemblies (GNP1/GNP2 and GNP1S/GNP2S) and (b) 17 nm IONP assemblies (IONP1/IONP2 and IONP1S/IONP2S). The *T*_m_ values are given in parentheses. (c-d) Extinction spectra of (c) GNP1S/GNP2S and (d) IONP1S/ IONP2S before and after *T*_m_.


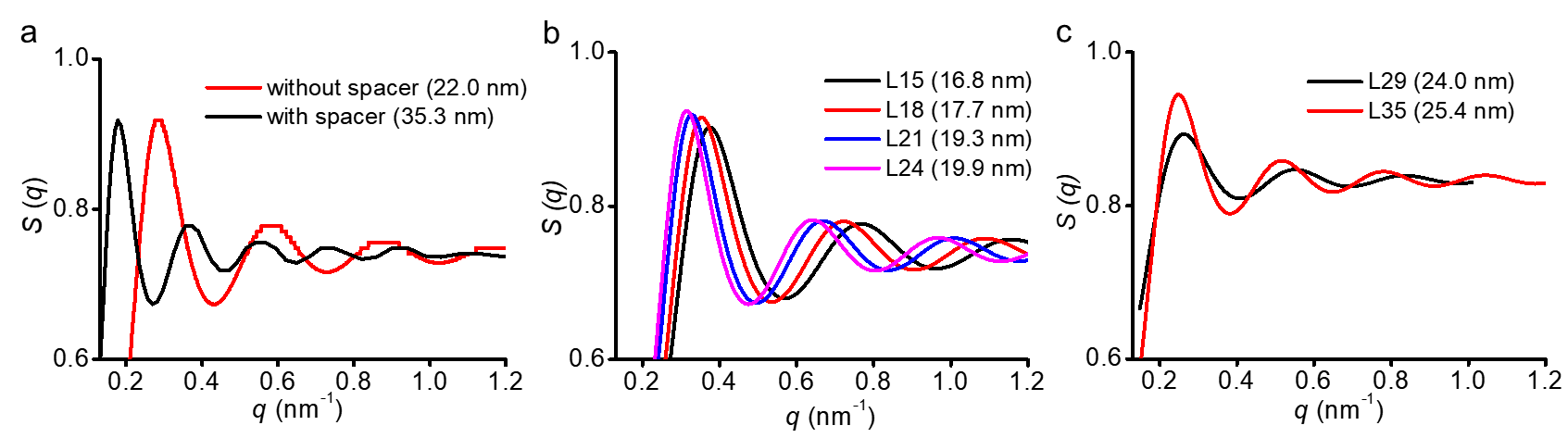


**Figure S14.** The structure factors of IONP assemblies from SAXS. (a) 17 nm IONP1S/IONP2S and IONP1/IONP2 formed with L21. (b) 13 nm IONP1/IONP2 formed with L15, L18, L21, or L24. (c) 13 nm IONP1S/IONP2S formed with L29 and L35.

**
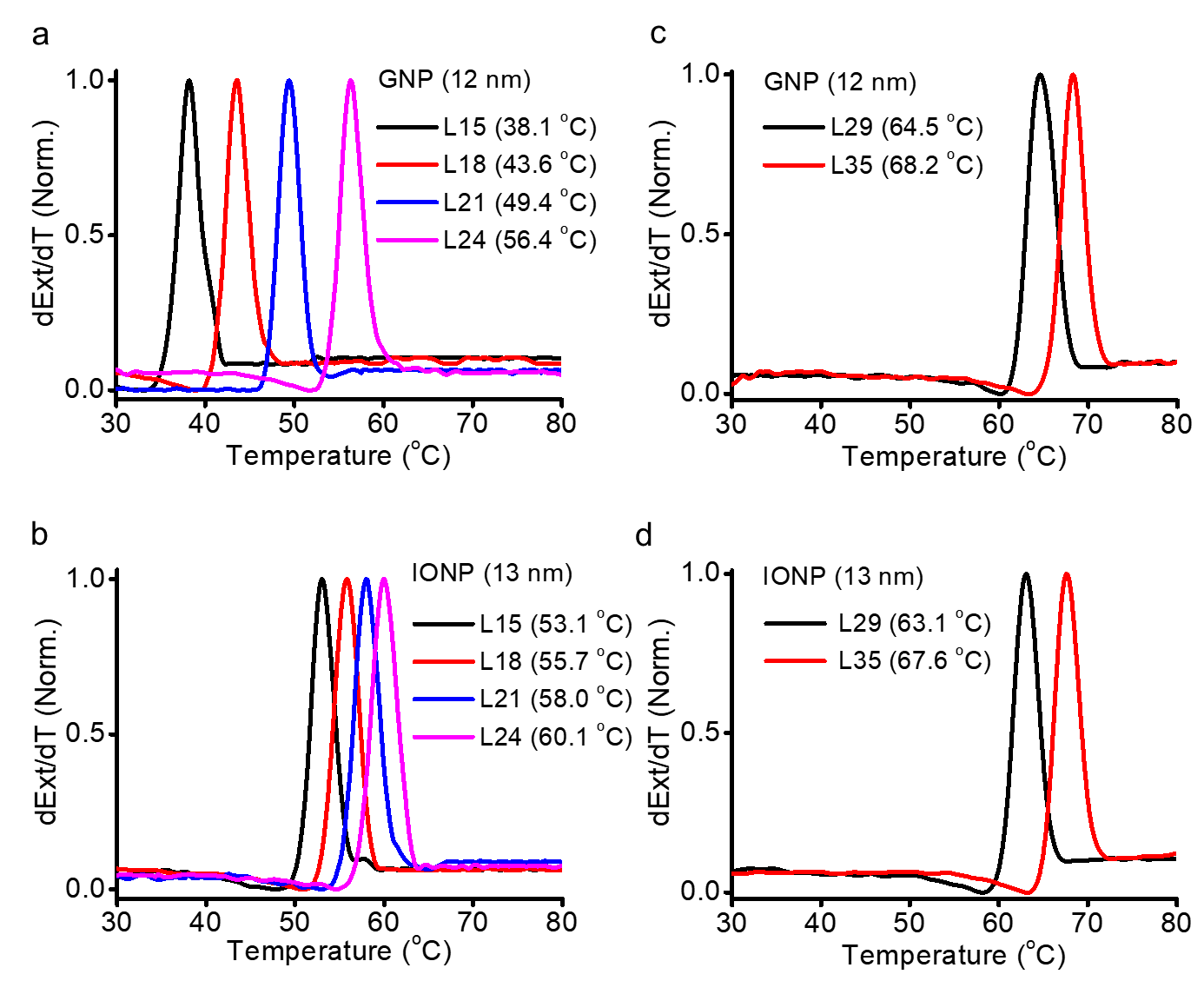
**

**Figure S15.** Thermal denaturation derivative plots of NP assemblies formed with L15-L35. (a) GNP1/GNP2 linked with L15-L24. (b) IONP1/IONP2 (13 nm) linked with L15-L24. (c) GNP1S/GNP2S linked with L29 and L35. (d) IONP1S/IONP2S (13 nm) linked with L29 and L35. The *T*_m_ values are given in parentheses.


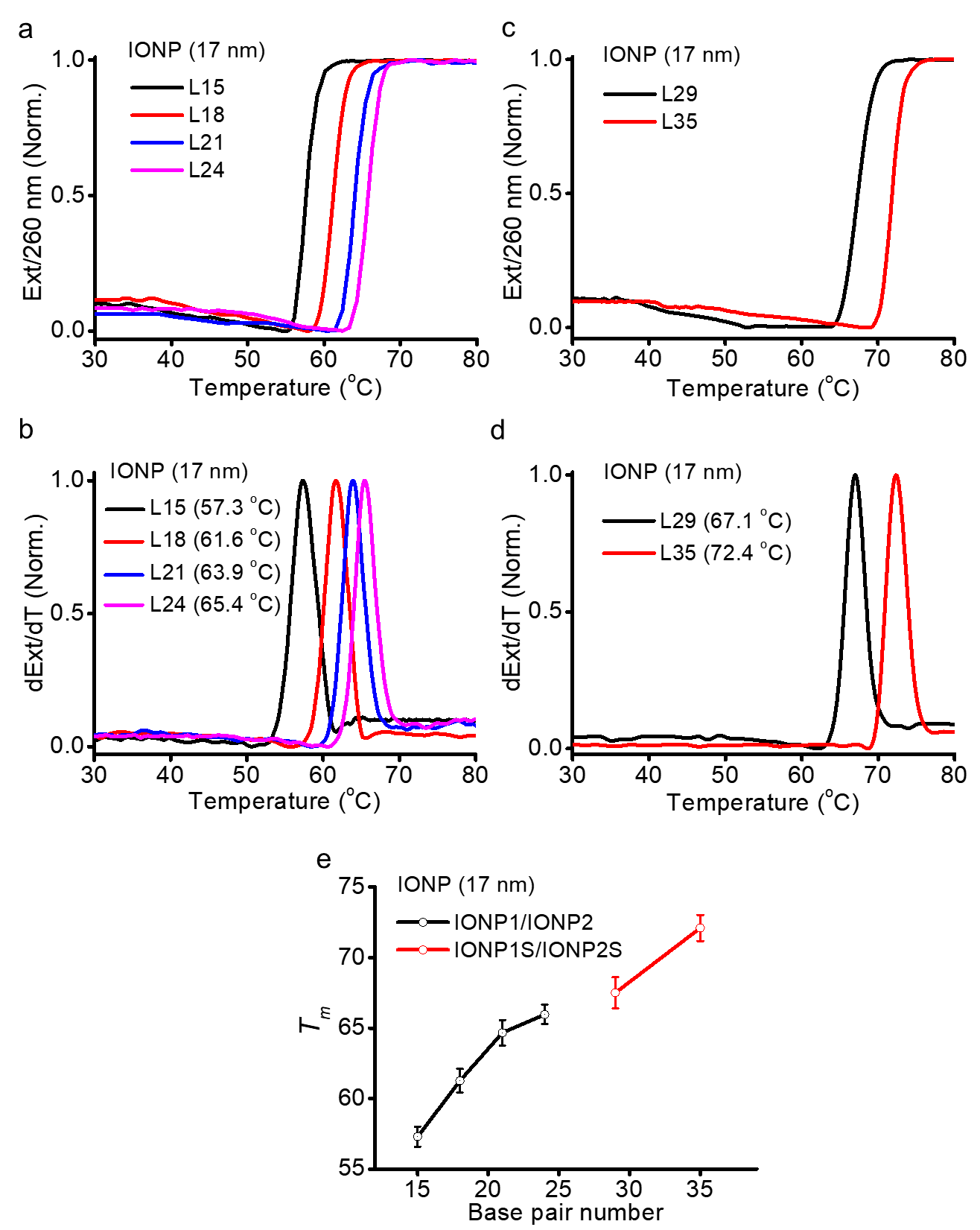


**Figure S16.** Thermal denaturation curves of IONP (17 nm) linked with L15–L35. (a-b) Melting profiles (a) and derivative plots (b) of IONP1/IONP2 prepared with L15-L24. (c-d) Melting profiles (c) and derivative plots (d) of IONP1S/IONP2S prepared with L29-L35. The *T*_m_ values are given in parentheses.

**III. References**

[1] B. Cheng, K. Ishihara, H. Ejima, Bio-inspired immobilization of low-fouling phospholipid polymers via a simple dipping process: a comparative study of phenol, catechol and gallol as tethering groups, *Polym. Chem.* **2020**, *11*, 249-253.

[2] S. K. Albert, H. V. P. Thelu, M. Golla, N. Krishnan, S. Chaudhary, R. Varghese, Self‐Assembly of DNA–Oligo(p‐phenylene‐ethynylene) Hybrid Amphiphiles into Surface‐Engineered Vesicles with Enhanced Emission, *Angew. Chem. Int. Ed.* **2014**, *53*, 8352-8357.

[3] J. J. Storhoff, R. Elghanian, R. C. Mucic, C. A. Mirkin, R. L. Letsinger, One-pot colorimetric differentiation of polynucleotides with single base imperfections using gold nanoparticle probes, *J. Am. Chem. Soc.* **1998**, *120*, 1959-1964.

[4] M. Park, S. Kang, C. Nam, K. Narasimha, W. B. Lee, S.-J. Park, Magnetic Field-Induced Self-Assembly of Conjugated Block Copolymers and Nanoparticles at the Air–Water Interface, *ACS Appl. Mater. Inter.* **2022**, *14*, 8266.

[5] P. J. Santos, R. J. Macfarlane, Reinforcing supramolecular bonding with magnetic dipole interactions to assemble dynamic nanoparticle superlattices, *J. Am. Chem. Soc.* **2020**, *142*, 1170.

[6] K. J. M. B., C. E. Wilmer, S. Soh, B. A. Grzybowski, Nanoscale forces and their uses in self-assembly, *Small* **2009**, 5, 1600.

[7] M. Galli, A. Guerrini, S. Cauteruccio, P. Thakare, D. Dova, F. Orsini, P. Arosio, C. Carrara, C. Sangregorio, A. Lascialfari, D. Maggioni, E. Licandro, Superparamagnetic iron oxide nanoparticles functionalized by peptide nucleic acids, *RSC Adv.* **2017**, *7*, 15500-15512.

[8] J. Mohapatra, F. Zeng, K. Elkins, M. Xing, M. Ghimire, S. Yoon, S. R. Mishrab, J. Liu, Size-dependent magnetic and inductive heating properties of Fe 3 O 4 nanoparticles: scaling laws across the superparamagnetic size, *Phys. Chem. Chem. Phys.* **2018**, *20*, 12879.
